# Secondary invasion: When invasion success is contingent on other invaders altering the properties of recipient ecosystems

**DOI:** 10.1002/ece3.3315

**Published:** 2017-08-17

**Authors:** Luke S. O'Loughlin, Peter T. Green

**Affiliations:** ^1^ Department of Ecology Environment and Evolution La Trobe University Bundoora Victoria Australia; ^2^ Present address: Fenner School of Environment and Society The Australian National University Canberra ACT, Australia

**Keywords:** facilitation, impacts of biological invasions, invasion complex, invasion success, invasional meltdown, population release, secondary spread, true entry

## Abstract

Positive interactions between exotic species may increase ecosystem‐level impacts and potentially facilitate the entry and spread of other exotic species. Invader‐facilitated invasion success—”secondary invasion”—is a key conceptual aspect of the well‐known invasional meltdown hypothesis, but remains poorly defined and empirically underexplored. Drawing from heuristic models and published empirical studies, we explore this form of “secondary invasion” and discuss the phenomenon within the recognized conceptual framework of the determinants of invasion success. The term “secondary invasion” has been used haphazardly in the literature to refer to multiple invasion phenomena, most of which have other more accepted titles. Our usage of the term secondary invasion is akin to “invader‐facilitated invasion,” which we define as the phenomenon in which the invasion success of one exotic species is contingent on the presence, influence, and impacts of one or more other exotic species. We present case studies of secondary invasion whereby primary invaders facilitate the entry or establishment of exotic species into communities where they were previously excluded from becoming invasive. Our synthesis, discussion, and conceptual framework of this type of secondary invasion provides a useful reference to better explain how invasive species can alter key properties of recipient ecosystems that can ultimately determine the invasion success of other species. This study increases our appreciation for complex interactions following invasion and highlights the impacts of invasive species themselves as possible determinants of invasion success. We anticipate that highlighting “secondary invasion” in this way will enable studies reporting similar phenomena to be identified and linked through consistent terminology.

## 1 INTRODUCTION

Understanding the mechanisms affecting the successful establishment, dominance, and spread (“invasion success”) of exotic species is central to developing effective management (Blackburn et al., 2011; Mack et al., 2000). Although empirical descriptions of invaders modifying recipient ecosystems to the benefit of other exotics are becoming more common (Adams, Pearl, & Bury, 2003; Green et al., 2011; Grosholz, 2005; Johnson, Olden, Solomon, & Vander Zanden, 2009; Simberloff & Von Holle, 1999), these interactions remain underexplored, poorly defined, and omitted from recognized heuristic models of invasion success (e.g., Blackburn et al., 2011; Catford, Jansson, & Nilsson, 2009). Properties of recipient ecosystems can be altered by the impacts of previously successful invaders, meaning that the presence and impact of those invaders should be considered a property of the recipient ecosystem to which subsequent invaders may respond. Successful invaders may facilitate the invasion success of others either alone or in concert (i.e., “invasional meltdown”; Simberloff & Von Holle, 1999) through direct or indirect mechanisms. This kind of phenomenon may be very common given the ubiquitous presence of invasive species in almost all ecosystems and, as such, requires formalization within the broader framework describing the determinants of invasion success.

The aim of this study is to define and explore the concept of invader‐facilitated invasion—sometimes referred to as “secondary invasion.” This is important because currently, invasion success facilitated by other invaders is not formally defined in the ecological literature and the term secondary invasion is increasingly being used interchangeably with others to describe a number of ecological phenomena. After first exploring these alternative uses and advocating the specific use of the term for invader‐facilitated invasions, we then use examples from the literature to discuss 1. how secondary invasion fits within the current recognized heuristic framework of the determinants of invasion success, 2. the mechanisms by which previously successful “primary” invaders may directly or indirectly facilitate secondary invaders via changes to the properties of recipient communities, 3. empirical case studies of secondary invasion that describe alternate pathways of secondary invasion based on which stage of the invasion pathway a species is facilitated from, 4. the kinds of data required to determine whether secondary invasion is occurring, and 5. how this definition and framework can inform research and management directions.

## 2 DEFINING “SECONDARY INVASION”

Increasingly, the term “secondary invasion” is being used to describe quite disparate invasion phenomena (Table 1), potentially creating confusion and lowering its heuristic value. The earliest use of the term in a broadly ecological context was by Wicklow, Bennett, and Shotwell (1987) to describe plant–pathogen dynamics in soybeans, where one fungal pathogen could only affect crops already infected by a different fungal pathogen (Wicklow et al., 1987). This example draws significant parallels to the phenomenon of “secondary infection” in humans, which is mostly well understood by nonspecialists to be where an initial pathogen weakens the body's immunity, permitting another pathogen to infect and cause harm where it otherwise would not (see definition in Soo, Khalid, Ching, & Chee, 2016). Since Wicklow et al.'s (1987) original use, “secondary invasion” has been applied to at least five unique invasion phenomena to emphasize temporal or spatial aspects of invasion dynamics, or the mechanism(s) permitting invasion (Table 1).

**Table 1:** Clarifying and differentiating the various uses of the term “secondary invasion”

Suggested term	Phenomenon
Secondary spread	Where an invasive species increases its range following initial invasion. This includes the invasion of an adjoining ecosystem and niche shifts, sometimes the product of rapid evolution. The geographic spread of round gobies into secondary waterways (Baldwin, Carpenter, Rury, & Woodward, 2012) and of the tree *Acer negundo* into previously uninvaded habitat (Erfmeier, Bohnke, & Bruelheide, 2011) are examples in which the term “secondary invasion” has been used in this way. This phenomenon is well defined within the invasion biology literature as “secondary spread” and should therefore not be substituted with the poorly defined “secondary invasion”
Secondary dispersal	Where a species increases its range due to further anthropogenic‐facilitated dispersal of propagules from a previously invaded location is also sometimes referred to as secondary invasion. This usage is applied most commonly to the spread of marine invaders, typically through ballast water, where genetic approaches are employed to determine the source population of new invasions (e.g., Albaina et al., 2016; Williams, Nivison, Ambrose, Dobbin, & Locke, 2015). However, “secondary spread” is also used to describe this phenomenon (e.g., Simkanin, Davidson, Falkner, Sytsma, & Ruiz, 2009). We suggest that secondary spread is misapplied here and that this situation has more in common with the recognized concept of “secondary dispersal” (vander Wall, Kuhn, & Beck, 2005)
Management‐mediated invasion	The invasion and increase in abundance of exotic species due to the anthropogenic management of another invasive species. Sometimes referred to as the “weed‐shaped hole” (Buckley, Bolker, & Rees, 2007), the term secondary invasion is used in this way almost exclusively when discussing exotic plants (Pearson, Ortega, Runyon, & Butler, 2016). For example, Gooden, French, and Turner (2009) described the secondary invasion of other exotics following management of the invasive woody shrub *Lantana camara*. This usage of the term describes situations in which human mediated facilitation has continued beyond the transport phase of invasion
Secondary Invasion	Invader‐facilitated invasion, where invasion success of one exotic species is facilitated by another exotic species. This usage is similar to the concept of “invasion complex” (D'Antonio & Dudley, 1993) which has not been widely adopted. We suggest that it is most appropriate to use the term “secondary invasion” in this context for reasons outlined in this paper. Examples of invasion success being dependent on the effects of other exotic species are increasingly being noted as increased attention is paid to facilitative interactions (Grosholz, 2005) and invasional meltdown (Green et al., 2011)
Sequential phase invasion	Used to describe an invasive species simply by sequence of arrival. This usage of the term “secondary invasion” to describe this phenomenon is very rare, but it should still be noted. Dietz and Edwards (2006) describe where the invasion success of the second species is not contingent on impacts of the first but rather species‐specific responses to new ecological circumstance—essentially defining a secondary invader as a species invading an ecosystem “second” due to chance arrival. This usage is poorly defined and provides little biological insight

All of the phenomena listed in this table have been described as “secondary invasions” within the scientific literature.

**Figure 1:**
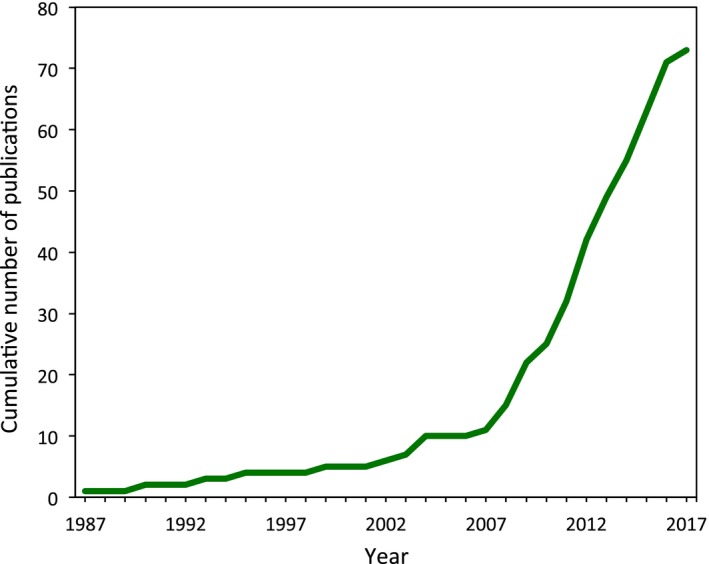
The cumulative number of publications in the ecological literature through time that specifically refer to “secondary invasion” in the title, keywords, and/or abstract. We searched the ISI Web of Science and Scopus databases in June 2017 for the term “secondary invasion” and constrained our results to the subject areas of environmental sciences and ecology, zoology, plant sciences, biodiversity conservation, marine and freshwater biology, entomology, and forestry. We identified 73 publications from 1987 onwards that used “secondary invasion” to mean any of the phenomena described in Table 1. Secondary invasion was used to mean “secondary spread” in the most publications (*n *=* *27), followed by “management‐mediated invasion” (*n *=* *16), secondary invasion as we have defined it here as “invader‐facilitated invasion” (*n *=* *14), “secondary dispersal” (*n *=* *12), and was used in an undefined way in a further four publications

Ecology is often bedevilled by conceptual imprecision and the inconsistent use of terminology (e.g., Cottee‐Jones & Whittaker, 2012; Peters, 1988), and while some ecologists think terminological prescription is either unnecessary or unachievable (e.g., Hodges, 2008, 2014; Jax & Hodges, 2008), inconsistent terminology can affect results (e.g., Fraser, Garrard, Rumpff, Hauser, & McCarthy, 2015) and slow scientific progress (Herrando‐Perez, Brook, & Bradshaw, 2014). With this in mind, we advocate for the very narrow use of the term “secondary invasion” to be applied in situations conceptually analogous to Wicklow et al.'s (1987) example in which one invader facilitates the invasion of another, in that case through the first invader's impact on the host plant. The problem is not trivial. We identified 73 publications since 1987 that have used the term in various contexts, with 85% of them appearing in the last decade alone (Figure 1). Clearly, the time is ripe to create a consistent definition for this emerging term.

Therefore, we define secondary invasion as the phenomenon in which invasion success of one exotic species (the secondary invader) is completely contingent on the presence, influence, and impact of one or more other exotic species (primary invaders). We define a primary invader as any exotic species that can successfully invade without the alteration of ecosystem properties by other exotic species. A secondary invader is defined as any exotic species that is unable to successfully invade due to some inhibiting property of the recipient ecosystem. A secondary invader only becomes so after primary invaders, through their presence and influence, alter that inhibiting property and invasion can proceed. We posit that this definition is the most appropriate and useful way to use the term as 1. the phenomenon we describe is distinct from other ideas of facilitated invasion and therefore requires formal definition, 2. using consistent terminology poses significant benefits for the synthesis of scientific knowledge, and 3. the definition of the word “secondary” and the way it is used in other fields means this phenomenon is the most accurate use of the term, compared to other ways it is currently used (Table 1).

Secondary invasion shares similarities with, yet remains distinct from, other concepts in the invasion literature, namely the invasional meltdown hypothesis (Simberloff & Von Holle, 1999), the passenger‐driver model (MacDougall & Turkington, 2005), and the lesser‐known “invasion complex” (D'Antonio & Dudley, 1993). Invasional meltdown is concerned with invasion success of two species being contingent on direct mutualistic interactions (Simberloff & Von Holle, 1999). If say a particular plant and insect were only able to invade with each other (pollinator‐resource limited), then that could be invasional meltdown. However, if the insect was a generalist pollinator and was able to reach high abundance without that particular plant, but the plant could only invade if that pollinator was present, then that would be secondary invasion. Invasional meltdown does predict an accelerating accumulation of exotic species as a consequence of invader–invader mutualism (secondary invasions); however, this was not the primary focus of the hypothesis and leaves secondary invasion with a restricted definition. The term secondary invasion was not used by Simberloff and Von Holle (1999) when introducing invasional meltdown, or when the concept was later refined (Simberloff, 2006). Both invasional meltdown and secondary invasion describe unique phenomenon in invasion ecology and propose distinct hypotheses.

Secondary invasion also draws parallels to the idea that invasive species are either “drivers” or “passengers” of change (active or inactive in the alteration of ecosystem properties) (MacDougall & Turkington, 2005). In principle, a primary invader would be a driver and a secondary invader would be a passenger. However, in the passenger‐driver model, passengers are a consequence of anthropogenic change and do not depend on drivers. Secondary invasion is similar as our driver (primary invader) is unrestricted and causes change, but quite distinct as our passenger (secondary invader) is never independent of our driver and the invasion process is highly interactive. Our concept draws the most similarities with “invasion complex” (D'Antonio & Dudley, 1993; —often erroneously cited as D'Antonio (1990)). D'Antonio and Dudley (1993) use two examples of soil alteration by successful invaders (increased nutrients and disturbance) leading to other invasions to define their concept as where “one invader takes hold, alters the physical habitat, and soon other exotic species are finding a new home.” Our definition of secondary invasion encompasses this idea, but broadens it to include direct, facilitative interactions between species and explicitly link changes in ecosystem properties to the invasion success of previously unsuccessful species.

## 3 SECONDARY INVASION WITHIN THE EXISTING CONCEPTUAL FRAMEWORK

**Figure 2:**
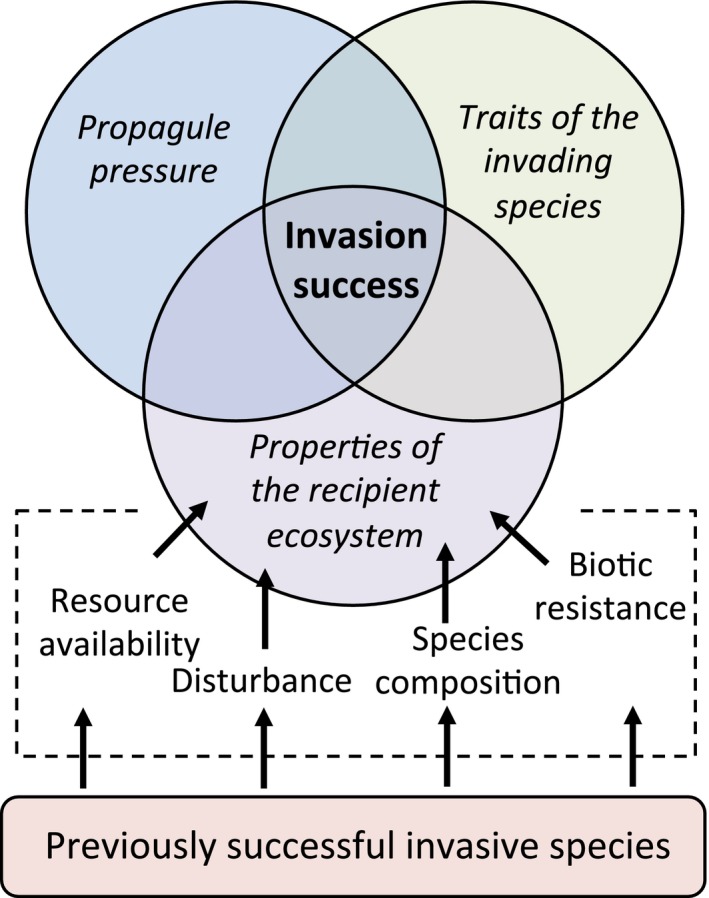
Conceptual framework outlining how propagule pressure, traits of the invading species, and properties of the recipient ecosystem interact to determine invasion success (modified from Catford et al., 2009). Each of these factors may be more or less important in determining a specific invasion event; favorable conditions in all three are required for a successful invasion. Specifically highlighted are the factors that contribute to the properties of the recipient ecosystem, as these are characteristics that can be altered by one or more previously successful invasive species (primary invader). Listed are only four examples of properties that primary invaders can affect, but any other biotic or abiotic characteristic of a recipient ecosystem can also be affected. Arrows indicate that primary invader(s) alter properties of the recipient ecosystem, which, in turn, contributes to determining the invasion success of subsequent invaders

The process of invasion is understood as a staged pathway in which different barriers must be overcome to move from one stage to the next (see Blackburn et al., 2011). The factors that facilitate a species to move from a recent introduction through to a highly abundant invasive (“invasion success”) will be the function of, and interaction among, propagule pressure, traits of the invading species, and properties of the recipient ecosystem (abiotic and biotic) (Catford et al., 2009) (Figure 2). When considering secondary invasion, we are interested in how the properties of a recipient ecosystem have initially provided a filter limiting invasion success, and how these properties can be altered by an exotic species (primary invader) in ways that permit additional (secondary) invaders to establish. Or alternatively, how one invasion changes the invasibility of a community, independent from the inherent invasiveness of the taxon, or the introductory dynamics.

Most conceptual frameworks that aim to explain or predict invasion success recognize the importance of environmental suitability for allowing species to enter, establish and spread (Blackburn et al., 2011; Catford et al., 2009; Heger et al., 2013; Leung et al., 2012; Rouget et al., 2016). However, the capacity for that suitability to change is rarely accounted for within these frameworks, and the mechanisms driving potential changes to properties of recipient ecosystems are seldom discussed. Robust quantitative frameworks developed to predict the risk of invasion (e.g., Rouget et al., 2016) will often use bioclimatic species distribution methods to determine environmental suitability, which do not account for biological interactions. Developing these kinds of frameworks is fundamental to broad management policy; however, the context‐specific nature of species invasions and inherent complexity of ecosystems means idiographic approaches remain necessary. Knowledge of the dynamics of the properties of recipient ecosystems is as important as understanding species traits and propagule pressure when predicting invasion success (Catford et al., 2009).

Properties of the recipient ecosystem important for determining invasion success include both abiotic and biotic characteristics (Blackburn et al., 2011; Catford et al., 2009). Exotic species will either be promoted or inhibited by one or more characteristics of an ecosystem, including (among others) resource availability, disturbance (type and frequency), and species composition (Boelman, Asner, Hart, & Martin, 2007; Levine, Adler, & Yelenik, 2004; Von Holle, Delcourt, & Simberloff, 2003; Walker & Vitousek, 1991) (Figure 2). However, complex interactions exist among these properties. Disturbance can drive resource availability (Mack et al., 2000), resource availability can determine species composition (Huston, 1979), and species composition can drive disturbance (Brooks et al., 2004). Therefore, any direct change caused by successful invaders has the potential to alter many other properties via indirect mechanisms (White, Wilson, & Clarke, 2006). We highlight the role of invaders in altering the properties of recipient ecosystems as the determinant of secondary invasion (Figure 2).

## 4 THE MECHANISMS BY WHICH PRIMARY INVADERS MAY FACILITATE SECONDARY INVADERS

**Figure 3:**
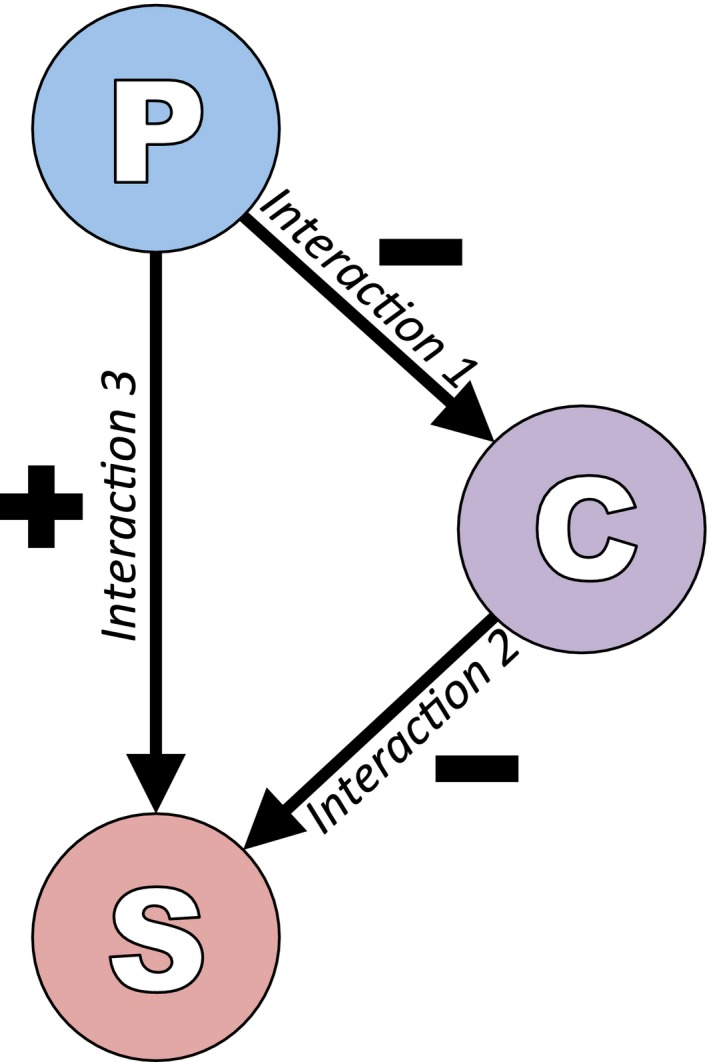
The secondary invasion interaction map. Primary invaders (P) that have a direct or indirect negative influence on a component (property) of the recipient ecosystem (C) [interaction 1], that has a direct or indirect negative influence on the invasion success of a secondary invader (S) [interaction 2], will therefore have an indirect positive influence on the invasion success of the secondary invader [interaction 3]. (S) could not invade the system without (P) changing (C). A direct interaction between (P) and (S) occurs when the presence of the primary invader provides a new (C) previously lacking in the community (i.e., the appropriate pollinator or disperser)

Primary invaders may indirectly facilitate secondary invaders by altering one or more properties of the recipient ecosystem that previously had a negative influence on a potential invader, thereby having an indirect positive influence on a secondary invader (Figure 3). Primary invaders may also directly facilitate secondary invasion by providing a unique property within the recipient ecosystem. The invasion literature contains many examples of 1. invasive species altering characteristics of an ecosystem (Figure 3 interaction 1), 2. properties of an ecosystem inhibiting invader success (Figure 3 interaction 2), and 3. invaders engaged in direct or indirect positive interactions with other species (Figure 3 interaction 3). The inherent complexity of species networks means all three interactions (Figure 3) are rarely explored together (see White et al., 2006).

Invasive species can alter biotic and abiotic properties of an ecosystem via a number of mechanisms. The most well‐known example of invasive species altering abiotic properties is the entry of nitrogen‐fixing plants into areas previously low in available N (Kourtev, Huang, & Ehrenfeld, 1999; Vitousek & Walker, 1989; Walker & Vitousek, 1991). The N‐enrichment of soils in Hawaii by the exotic shrub *Myrica faya* promotes the establishment of previously inhibited plant species (Walker & Vitousek, 1991) and facilitates higher densities of exotic earthworms (Kourtev et al., 1999). Nitrification of the soil increased the invasibility of the community. Invasive species may also alter key aspects of disturbance regimes and change competitive networks. For example, invasion by a pasture grass *Andropogon gayanus* in northern Australia increases fire intensity, thereby transforming species‐rich native savannah to exotic‐dominated grassland (Setterfield et al., 2013). The establishment of the exotic shrub *Mimosa pigra* in tropical Australia alters the vertebrate abundances through stand dominance and altered vegetation structure (Braithwaite, Lonsdale, & Estbergs, 1989). These examples demonstrate both direct and indirect mechanisms by which primary invaders can alter properties of ecosystems, thereby changing the invasibility of that community for others (Figure 3 interaction 1).

In many cases, properties of the recipient ecosystem will inhibit species invasion, examples of which form the basis of the biotic resistance model (Levine et al., 2004). A key mechanism that allows ecosystems to deter potential invaders is the presence of competitively dominant species (Huang, Carrillo, Ding, & Siemann, 2012; Levin, Coyer, Petrik, & Good, 2002; Maron & Vila, 2001). In the Gulf of Maine, the exotic green alga *Codium fragile* was only able to invade following the removal of the competitively dominant native kelp (Levin et al., 2002). *Codium fragile* could only establish following the creation of a gap in the kelp forest, mediated by another invasive species growing epiphytically on the kelp (Levin et al., 2002). Potential invaders may also be inhibited by multiple properties of recipient ecosystems (Cross, 1981; Morales & Aizen, 2002; O'Loughlin & Green, 2016). For example, Morales and Aizen (2002) demonstrated that exotic plants were facilitated directly by increased disturbance and indirectly by increased presence of exotic insect pollinators in disturbed areas. These species were (to an extent) both pollinator‐limited and competitively inferior, meaning invasion would not be successful until disturbance changed competitive networks and increased pollination services. Any species directly altering properties of recipient ecosystems has the potential to indirectly facilitate a previously inhibited species (Figure 3 interaction 2).

Exotic species can also form strong associations that will directly facilitate persistence and prevalence within a community (Barthell, Randall, Thorp, & Wenner, 2001; Constible, Sweitzer, Van Vuren, Schuyler, & Knapp, 2005; Ricciardi, Whoriskey, & Rasmussen, 1997). Common direct facilitative interactions include pollination, such as exotic honey bees increasing seed set of an exotic plant (Barthell et al., 2001), and dispersal, such as introduced bison increasing the spread of seeds of exotic plants (Constible et al., 2005). Direct mutualistic relationships between invasive ants and honeydew‐secreting insects are common, resulting in significant population increases of both (Bach, 1991). An indirect effect of this kind of mutualism is that high ant densities have a positive effect on plants through removal of herbivorous insect larvae and excess honeydew (Bach, 1991). These direct interactions between primary and secondary invaders demonstrate an alteration of the properties of an ecosystem through the inclusion of something previously absent (Figure 3 interaction 3). All of these examples highlight the amount of research effort required to understand complex interactions in determining the mechanism of invasion success.

## 5 DEMONSTRATED SECONDARY INVASIONS AT DIFFERENT STAGES OF THE INVASION PATHWAY

**Figure 4:**
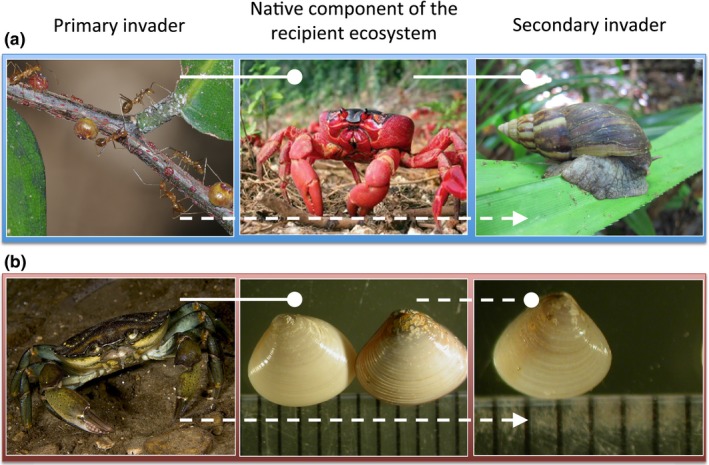
Two examples of interacting organisms and how the invasion success of the secondary invader is contingent on the presence of a primary invader modifying a native component of the recipient ecosystem. (a) Mutualism between invasive yellow crazy ants and scale insects removes the native red crab which allows entry into the community by the giant African land snail that were previously predated upon by the native crab (Green et al., 2011). (b) The invasive green crab preferentially predates the native *Nutricola* clam, allowing the population release of the exotic clam, *Gemma gemma*, which was competitively inferior to the native clams (Grosholz, 2005). Solid and dashed lines denote direct and indirect interactions respectively. Circles and triangles denote negative and positive interactions, respectively. Clam photographs include ruler for scale (1 mm between lines). All pictures in example (b) by Dr E Grosholz

**Figure 5:**
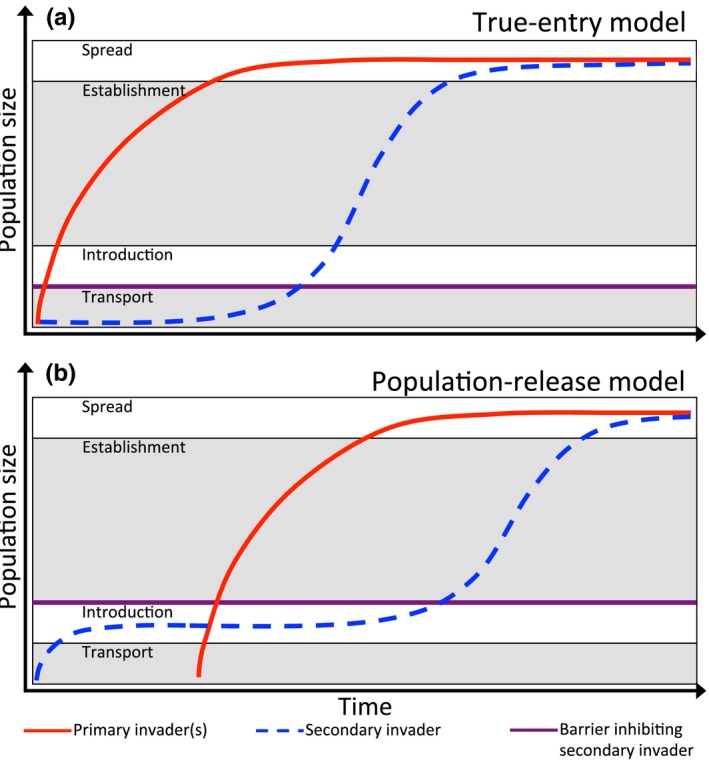
Models of secondary invasion that show the population growth of invaders over time in relation to the stages of the invasion pathway (see Blackburn et al., 2011). In both models, the secondary invader will not increase until the primary invader has reached the final stage along the invasion pathway (spread), at which point its impacts have altered properties of the recipient ecosystem. The models differ in which early stage of the invasion pathway the secondary invader is unable to move past. For the true‐entry model, the secondary invader cannot progress past the transport stage until the primary invader has altered the ecosystem (barrier prohibits introduction). For the population‐release model, the secondary invader has persisted in the community but is unable to progress past the entry stage until a primary invader is introduced and alters the properties of the ecosystem (barrier prohibits establishment)

One clear empirical example supporting the concept of secondary invasion as we define it comes from Christmas Island, a remote oceanic island in the Indian Ocean. Invader–invader mutualism between introduced yellow crazy ants (*Anoplolepis gracilipes*) and honeydew‐secreting scale insects leads to positive population‐level feedback resulting in high‐density supercolonies of ants that accelerate and diversify impacts across the rainforest ecosystem (Abbott, 2006; O'Dowd, Green, & Lake, 2003). The abundant native omnivore–detritivore and ecosystem engineer, the red land crab (*Gecarcoidea natalis*), is completely extirpated in supercolonies where ants overwhelm and kill the crabs (O'Dowd et al., 2003). This deletion increases resources by altering seedling recruitment dynamics and leaf‐litter breakdown, and removes a potential predator (Lake & O'Dowd, 1991; O'Dowd et al., 2003). The entry and spread of the exotic giant African land snail (*Achatina fulica*) into rainforest on the island has been facilitated by these impacts (Green et al., 2011). Modeling seven years of incidence data showed the probability of land snail invasion was facilitated 253‐fold in ant supercolonies, but invasion was completely impeded in uninvaded forest where predaceous crabs remained abundant (Green et al., 2011). The red land crab acted as a filter that inhibited the entry of these exotic snails into the rainforest. In this case, the primary invaders had altered the recipient ecosystem by disrupting biotic resistance and providing enemy‐free space, allowing the giant African land snail to pass through that environmental barrier and move from the transport to introduction stage along the invasion pathway (Figure 4a; Figure 5a).

A second empirical example comes from Bodega Bay Harbor in California. A predatory green crab, *Carcinus maenas*, invaded the ecosystem and caused fivefold to 10‐fold declines in native clam (*Nutricola* spp.) abundance (Grosholz et al., 2000). Also present was the exotic eastern gem clam (*Gemma gemma*), which had been persisting in low abundance since at least the 1960s, and then began to increase rapidly in abundance following the introduction of the green crab (Grosholz, 2005). Predation and competition experiments revealed that green crabs preferentially consumed native clams and that high natural densities of native clams had strong interspecific competitive effects on *G. gemma* (Grosholz, 2005). Native clams had inhibited *G. gemma* from establishing an invasive population through competitive dominance. In this case, the primary invader had altered the recipient ecosystem by reducing the abundance of a competitively dominant species, allowing a species to overcome that barrier and move from the introduction to establishment stage along the invasion pathway. (Figure 4b; Figure 5b).

Based on these examples, we propose two distinct pathways by which secondary invasion can occur: the “true‐entry” and the “population‐release” models (Figure 5). Our two models are distinguished by the stage at which a secondary invader is facilitated. The true‐entry model describes the situation in which an exotic species alters the recipient community to allow another exotic to move from the “transport” to “entry” stage and onwards (i.e., Green et al., 2011) (Figure 5). Alternatively, the population‐release model describes the facilitated move from “entry” to “establishment” and becoming invasive (i.e., Grosholz, 2005) (Figure 5). In principle, a third model of secondary invasion could exist whereby a species is facilitated from “establishment” to the “spread” stage of the invasion pathway (Blackburn et al., 2011). We could not find an empirical case study of secondary invasion to support this third model, potentially because much research considers a highly abundant established species to already be “invasive,” regardless of their capacity to spread.

These two studies also highlight a key consequence of leaving the phenomenon of invader‐facilitated invasion without formal definition. Despite being the strongest examples of secondary invasion, only Green et al. (2011) specifically used that term with Grosholz (2005) instead described his findings as an “accelerated invasion.” More examples of secondary invasion as we describe it have been published in recent years, including research on exotic land snails (O'Loughlin & Green, 2015, 2016), soil seed banks (Gioria, Dieterich, & Osborne, 2011; Gioria, Jarošík, & Pyšek, 2014; Gioria & Pyšek, 2015; Gioria, Pyšek, & Moravcova, 2012), and invasive plants (Flory & Bauer, 2014; French, 2012; Stotz, Gianoli, Patchell, & Cahill, 2017); however, these are only the studies that have explicitly used the term “secondary invasion” (see Figure 1). It is likely that other examples of invader‐facilitated invasion have been published in that time, but our ability to identify them is constrained by the absence of consistent and clear terminology for the phenomenon.

## 6 THE COMPLEXITY OF DETERMINING SECONDARY INVASION

Species distribution, niche models, environmental suitability, and species life history trait data are widely used to predict invasive range and impacts of exotic species, to develop quantitative risk assessment frameworks, and occasionally to infer the mechanism of invasion success (Leung et al., 2012; Rouget et al., 2016; Václavík & Meentemeyer, 2009). These models are powerful tools for researchers, managers, and policymakers to most appropriately direct resources and effort to minimize the impacts of biological invasions (Pheloung, Williams, & Halloy, 1999). However, our clear examples of secondary invasion (Green et al., 2011; Grosholz, 2005) came from meticulous on‐ground observations and detailed field studies and experiments involving complex systems. Although giant African land snails were unable to invade the rainforests of Christmas Island prior to the extirpation of the native crab (Green et al., 2011), elsewhere within their exotic range they are prominent primary invaders (Cowie, Dillon, Robinson, & Smith, 2009; Thiengo, Faraco, Salgado, Cowie, & Fernandez, 2007). Similarly, only after years of studying the community wide impacts of the introduced green crab (Grosholz et al., 2000) did the results of indirect facilitation on another exotic species become evident (Grosholz, 2005). The intricacies of these interactions suggest predicting secondary invasions through modeling techniques based on species identity and static information of habitat suitability alone may not be possible.

Distinguishing our alternative models of secondary invasion is limited by whether we can confidently determine species absence. As failure to detect does not necessarily mean species absence, techniques for determining the survey effort required to confidently record absence are increasingly important (McCarthy et al., 2013). Species abundance obviously influences detectability, and new models based on time to detection of the first individual provide a way to scale detection rates to cases of low abundance when direct estimation is impractical (McCarthy et al., 2013). Understanding which invasion stage a species is in at any point plays an important role in risk assessment and quantifying invasion risk (Leung et al., 2012; Rouget et al., 2016). Similarly, these tools would be particularly important when determining at which stage of the invasion pathway a secondary invader was facilitated from and therefore distinguishing true entry from population release.

Another potential challenge is distinguishing between facilitated population release and simply a lag in population establishment. Introduced species often persist in small populations for extended periods before rapidly increasing in abundance (Groves, 2006), thought to be due to species requiring time to build up the necessary propagule pressure to dominate and spread (Catford et al., 2009; Richardson et al., 2000). However, there are several biological and environmental mechanisms that underlie lag phases beyond simply population growth processes, as well as observed lag phases potentially being a statistical artifact or result of inconsistent sampling effort (Aikio, Duncan, & Hulme, 2010; Essl et al., 2015). The invasion of *Mimosa pigra* in tropical Australia has been widely cited as a textbook case of a lag in invasion (Braithwaite et al., 1989; Lonsdale, 1993). This exotic shrub persisted as a benign introduction for a century before dramatically increasing in abundance and becoming a high impact invader (Braithwaite et al., 1989). Exotic water buffalo (*Bubalus bubalis*) established in the area around the same time as *Mimosa* began to spread (Lonsdale, 1993), and although Lonsdale (1993) concluded the spread of *M. pigra* spread was due to dispersal by floodwater, there was a strong correlation between the rate of *M. pigra* spread and buffalo numbers. This association begs the question of whether *M. pigra* invasion is a classic example of a time lag in invasion, or rather that of positive interactions between exotic species and therefore a population‐release secondary invasion?

Hypothesizing that the ecosystem changes associated with one invasive species could have a facilitative effect on the invasion success of another is the first and easiest step in an investigation of secondary invasion. If the entry and spread of an exotic species is rapid, it may be difficult to determine whether the species was facilitated through secondary invasion mechanisms. Similarly, a period of low density prior to any change can be indicative of a facilitated release but not always so. To demonstrate and predict secondary invasion, quality data are required for 1. species time of arrival, 2. population size and dynamics, 3. species interactions and influence on the ecosystem, and 4. how the community has changed functionally and structurally over time. We suggest that the scientific, management, and policy benefits of the ecological understanding attained following a detailed investigation of secondary invasion would offset the apparent difficulties and challenges in attempting demonstrate the phenomena.

## 7 INFORMING INVASIVE SPECIES MANAGEMENT AND RESEARCH

By clarifying and redefining the concept of secondary invasion, we have provided the language, heuristic framework, and empirical tools for researchers and managers of biological invasions to test hypotheses, discuss findings, and prioritize actions. Secondary invaders are an indirect result of other invasion processes that have increased the invasibility of a community. Through understanding the mechanism behind the invasion success of particular species (primary v secondary invaders), managers should be able to make more informed decisions on which actions will maximize ecological benefit. For example, where primary invaders have been controlled on Christmas Island, secondary invaders have been indirectly controlled following the reestablishment biotic resistance in the community (Green et al., 2011; O'Loughlin & Green, 2015). Prioritizing the management of a secondary invader will not control the mechanism that altered the invasibility of the community in the first instance and could be an inefficient use of resources.

Uncertainty exists throughout the invasion process (Leung et al., 2012), and our concept definition aims to assist researchers and managers to minimize this. As defined here, secondary invasion 1. removes linguistic uncertainty by introducing consistent and appropriate terminology, 2. addresses stochasticity by emphasizing the need to account for abiotic and biotic changes in recipient ecosystems, and 3) aims to lower epistemic uncertainty by advocating the need for detailed empirical investigations in order to understand these biotic indirect effects. The documentation of complex interactions is at the forefront of ecological research, and our proposed models of secondary invasion aim to provide clarity of context for future researchers to frame their findings and discuss their work. As the complete eradication of invasive species is rarely feasible, understanding secondary invasion as a function and potential outcome of novel ecosystems is important for both policy and management (Hobbs et al., 2006). Our secondary invasion framework has implications for the development of invasion biology theory by identifying how previously successful invaders can influence the invasion success of others, as well as aiding in the prediction of future problem species for conservation management.

## CONFLICT OF INTEREST

None declared.

## AUTHORS CONTRIBUTIONS

LSO and PTG developed the conceptual framework of this research. LSO took lead on reviewing the literature and preparing the manuscript. PTG contributed significantly to developing the ideas, analysis and interpretation, and revision of manuscript drafts.
